# The Protective Effect of Flavonoids in the Diet on Autophagy-Related Cardiac Impairment

**DOI:** 10.3390/nu16142207

**Published:** 2024-07-10

**Authors:** Serena L’Abbate, Claudia Kusmic

**Affiliations:** Istituto di Fisiologia Clinica, Consiglio Nazionale delle Ricerche (CNR), 56124 Pisa, Italy; serenalabbate@cnr.it

**Keywords:** flavonoids, healthy diet, cardiovascular disease, macroautophagy, preclinical models

## Abstract

The compounds known as flavonoids, commonly found in fruits, vegetables, legumes, medicinal herbs, chocolate, and coffee and tea beverages, have been extensively researched for their impact on cardiovascular health. Flavonoids, with their demonstrated potential, have shown promising effects in regulating blood vessel function and apoptotic processes, as well as in improving lipid profiles. While their powerful antioxidant properties were initially thought to be the main reason behind these effects, recent studies have uncovered new insights into the positive effects of flavonoids on cardiovascular health, and researchers have now identified several signaling pathways and mechanisms that also play a role. Of particular interest are the studies that have highlighted the role of autophagy in maintaining the physiological functions of cardiomyocytes and protecting them from harm. Recent publications have linked the dysregulation of autophagic processes with the development of cardiomyopathies, heart failure, and other cardiovascular diseases. This review aims to present the latest, novel findings from preclinical research regarding the potential beneficial effects of flavonoids on various heart conditions associated with altered autophagy processes.

## 1. Introduction

Good nutrition is essential for maintaining good health and is considered one of the top risk factors that, when controlled, can significantly reduce the risk of death and disability worldwide [1]).

Recently, a clinical study conducted on data from about 250,000 adults from 80 countries provided a robust and consistent association between the consumption of a diet comprising higher amounts of fruits, vegetables, nuts, legumes, and a moderate amount of fish and whole-fat dairy and a lower risk of cardiovascular disease (CVD) and mortality in all regions of the world [2]. These six healthy components, considered protective foods, are mainly in line with those recommended by modern nutrition science and dietary guidelines worldwide. Interestingly, the study also found that the risks of death and vascular events were higher in adults with inadequate intake of protective foods. However, these dietary components are often disregarded by the public’s daily practice. Therefore, it is crucial to elucidate the diversity of compounds and nutrients in these foods that provide heart-protective properties and the cellular mechanisms underlying their beneficial effects.

Amidst the vast array of phenolic compounds, flavonoids are a promising cardioprotective group. Numerous preclinical studies have highlighted their pleiotropic effects on multiple risk factors and pathways relevant to cardiometabolic diseases [3]. With over 5000 naturally occurring flavonoids identified to date, their variable bioavailability after food intake, and the growing evidence of newly discovered signaling pathways involved, the emerging complexity of the role of flavonoids in supporting cardiovascular health is fascinating. These findings hint at the untapped potential of flavonoids, sparking further curiosity and research in this field.

Phytogenic flavonoids affect the physiological and pathological processes of CVD by impacting multiple biochemical signaling pathways and cellular functions. Some of these, such as antioxidant and anti-inflammatory mechanisms, have been extensively studied. Recently, scientific attention has turned to the disruption of the cellular process of autophagy in the context of CVD development [4]. However, the cross-talk between autophagy-related CVD and flavonoids remains still largely unexplored.

This review delves into the state-of-the-art preclinical research findings, shedding light on the impact of flavonoids on autophagy, a crucial cellular function necessary for the proper functioning of the cardiomyocyte and, therefore, the heart. We first summarize the role of autophagy in maintaining cellular and mitochondrial homeostasis and the relevant molecular mechanisms and signaling pathways affected by pathophysiological conditions. Additionally, we explore how flavonoids can help regulate autophagy in preclinical CVD models. By understanding how dietary flavonoids can modulate autophagy in a dysfunctional heart, nutraceutical strategies can be tailored to improve patient outcomes by intervening specifically in the autophagy process.

## 2. Mitochondrial Homeostasis in the Heart

The heart, a vital organ, is one of the body’s most energy-demanding tissues, constantly requiring adenosine triphosphate (ATP) to maintain normal cellular processes and proper organ function. A mismatch between energy supply and demand can lead to heart failure and cardiac dysfunction. With high energy demands, the heart is a powerhouse of mitochondria, estimated to make up about one-third of its total mass and produce 6–30 kg of ATP daily [5]. This staggering energy production underscores the heart’s critical role in our health. As cardiomyocytes perform day-to-day metabolic functions at a high rate, they generate and are exposed to high amounts of reactive oxygen species (ROS) and other damaging or toxic molecules. Over the cardiomyocyte’s lifetime, this excessive ROS production can disrupt mitochondrial metabolism, significantly impacting their health and, consequently, the heart’s function. The heart mainly comprises terminally differentiated cardiomyocytes, which cannot divide to dilute the ROS toxicity from the mitochondria. Therefore, the heart heavily depends on the efficient and coordinated mechanisms of mitochondrial quality control that also regulate the relative stability of mitochondrial quantity.

Mitochondrial quality control, a complex and vital process, involves three key components: mitochondrial biogenesis, mitochondrial dynamics (fusion and division), and mitophagy [6]. Mitochondrial biogenesis is responsible for regulating the mitochondrial mass in the cell. Mitophagy, mediated by different receptors, helps regulate the renewal and degradation of dysfunctional mitochondria. Additionally, the coordinated processes of mitochondrial fusion and division play a crucial role in repairing damaged mitochondria. The mitochondrial dynamics help maintain the pool of mitochondria within a cell and optimal oxidative phosphorylation (OXPHOS) activity by allowing efficient transport and distribution of mitochondrial content. On the one hand, mitochondrial fission separates daughter cell mitochondria with damaged membrane potential from healthy mitochondria [7]. On the other hand, the fusion process allows the exchange of gene products and metabolites between the fusing mitochondria to enhance their overall respiratory function. Mitophagy, a specific form of autophagy, is a highly conserved housekeeping process that helps eukaryotic cells maintain mitochondrial quality control and prevents stress by degrading unnecessary or dysfunctional components of organelles through lysosome-dependent mechanisms [8,9]. In the physiological setting, these processes are active in cardiomyocytes to warrant cellular homeostasis. However, they are critical under stress conditions where molecular ageing and poor mitochondrial quality control can lead to cardiovascular diseases. Cardiovascular disorders in which mitochondrial dysfunction is relevant include, but are not limited to, ischemic heart disease, hypertension, cardiac hypertrophy, and heart failure. Understanding and researching these mechanisms is paramount in our quest to unveil new interventional strategies to face CVDs.

### Selective Autophagy Helps in Maintaining Cellular Stability

Maintaining the balance of energy metabolism and the stability of the cell environment, as well as regulating cell renewal, growth, and development, are crucial physiological processes for a living organism. Autophagy is a process that involves three different types—microautophagy, chaperone-mediated autophagy, and macroautophagy. Each type follows a different pathway to deliver cargo to lysosomes. Microautophagy is a process in which lysosomes directly engulf cytosolic components by invaginating their membrane. The vacuole containing the cargo then separates from the membrane and becomes internalized within the lysosome, forming a microautophagic body broken down by vacuolar hydrolases into macromolecules that can be recycled [10].

Chaperone-mediated autophagy is a process that eliminates unwanted cytosolic proteins without involving lysosomal invagination. Instead, chaperone proteins such as heat shock cognate 70 protein (HSC70) recognize cytosolic cargo marked for degradation by their consensus sequence, known as the KFERQ-like motif. The chaperone–cargo complex then associates with lysosomal-associated membrane protein-2A (LAMP-2A), bound to the lysosomal membrane. As a result, the unfolded cytosolic protein is translocated into the lysosome and subsequently degraded [11].

**Figure 1:**
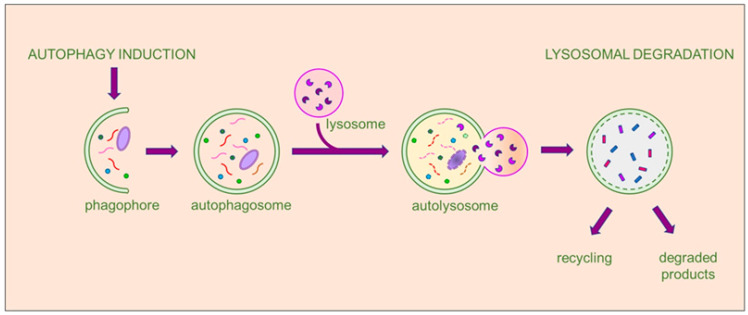
The multistep process of macroautophagy. When autophagy is activated, the cellular process begins with the engulfment of the autophagic cargo by a double membrane, initiating the formation of a cup-shaped structure called a phagophore. The phagophore then expands and transforms into a double membrane vesicle, developing into the autophagosome. Subsequently, the autophagosome fuses with lysosomes to form the autolysosome, where the autophagic cargo undergoes degradation by the hydrolytic enzymes of the lysosome and recycling of the degradation products. The entire process is tightly regulated by a multitude of proteins and factors at each stage. The final products may be recycled, if useful to the cell, or eliminated.

During macroautophagy, cytosolic proteins and organelles are sequestered into a double-membrane vesicle called autophagosome. This vesicle then fuses with the lysosome to form an autolysosome. The contents of the autolysosome are then degraded, allowing for recycling and the generation of ATP. Hereafter, macroautophagy is referred to as autophagy, which has been most extensively studied. Autophagy involves the following steps: process induction, vesicle nucleation, vesicle elongation and autophagosome formation, and retrieval and fusion between autophagosomes and lysosomes, as shown in Figure 1.

Although autophagy was initially perceived as a nonselective degradation process that engulfs and recycles cytoplasm material to provide energy and raw byproducts, increasing evidence indicates that autophagy can operate in a highly selective manner, according to various specific pathways. Selective autophagy targets specific types of cargo for degradation, such as damaged organelles (mitophagy, pexophagy, lysophagy, ER-phagy, ribophagy), aggregated proteins (aggrephagy), or invading bacteria (xenophagy) [12,13,14,15,16,17]. Not surprisingly, aberrant selective autophagy has been associated with various human pathologies [18,19,20]. The formation of autophagosomes is a multistep process mediated and regulated by autophagy-related (ATG) proteins, which are conserved from yeast to mammalian cells. Over 40 ATG genes have been identified, with most necessary for nonselective and selective autophagy [12,17,21]. The process is regulated by crucial upstream regulators such as the nutrient sensors mTOR (mechanistic target of rapamycin) and AMP (adenosine monophosphate)-activated kinase (AMPK), which, in turn, regulate the phosphorylation of Unc-51 kinase-like-1 (ULK-1), a kinase that initiates autophagy [22]. Selective removal of autophagic cargo is a complex process extensively reviewed elsewhere [20,23]. We will briefly summarize the primary phases. The process requires the tagging of cargo with specific marker proteins by ubiquitylation. Autophagy receptors recognize these markers and facilitate cargo degradation by binding to members of MAP1LC3/LC3 (microtubule-associated protein 1 light chain 3) and GABARAP (gamma-aminobutyric acid (GABA) type A receptor-associated protein) subfamilies. These subfamilies are anchored to autophagosomal membranes to promote cargo recruitment to the phagophore [24]. Autophagic receptors can interact with phagophore-anchored LC3/GABARAP proteins as they possess a short linear LC3-interacting region (LIR) motif. Prominent examples of autophagy receptors are protein p62 (also known as SQSTM1) and optineurin (OPTN). Several types of receptors or receptor-related factors have been identified in mammals that recognize mitochondria tagged for degradation, including BCL-2/adenovirus E1B 19kD protein-interacting protein 3 (BNIP3), FUN14 Domain Containing 1 (FUNDC1), PTEN-induced kinase 1 (PINK1), Parkin RBR E3 ubiquitin-protein ligase (Parkin), and BCL2 Like 13 (BCL2L13).

Due to the abundance of mitochondria in cardiomyocytes, mitophagy plays a crucial role in the heart’s physiology and pathophysiology. As a result, the function and mechanism of mitophagy in cardiac diseases have gained significant attention since the early 21st century [25,26,27]. Studies have shown that mitophagy is essential for degrading damaged or unnecessary mitochondria in cardiomyocytes at baseline and in response to stress [28,29]. Various stressors, such as ischemia, hypoxia, oxidative stress, and metabolic dysregulation, can negatively affect autophagy and/or mitophagy. This, in turn, has the potential to adversely impact cardiac function and cause CVD. Interestingly, autophagy plays protective and detrimental roles, and both the loss and exacerbation of different forms of autophagy can lead to the development of CVD [27,30].

It has been described that autophagy is triggered in cardiomyocytes during stress, hemodynamic overload, and acute ischemic insult. It has also been observed that autophagic activity is inversely related to postinfarction cardiac remodeling and dysfunction [31]. This suggests that activation of autophagic machinery is a compensatory response to increase the energy supply to meet the cellular demand during excessive wall stress and hypoxia, which can cause a low-energy state in cardiomyocytes. It is now broadly accepted that, during chronic ischemic remodeling, autophagy activation exerts protective effects [30,32,33]. However, the role of autophagy in the acute phases of reperfusion is still largely unclear and debated. There is evidence that the upregulation of autophagy increases reperfusion injury and, consistently, its inhibition is beneficial [34,35]. Nevertheless, the pharmacological induction of cardiomyocyte autophagy has proven to be effective in blunting ischemia/reperfusion (I/R) injury [36].

There is conflicting evidence on the role of autophagy in chemotherapy-induced cardiomyopathy, too. Several studies have concluded that autophagy has a dual effect on the treatment and progression of doxorubicin (DOX)-induced cardiotoxicity. Some studies suggest that DOX increases cardiac autophagic action, which in turn leads to cardiotoxicity [37,38,39]. Other studies, however, suggest that DOX induces cardiotoxicity by inhibiting autophagy [40,41]. Interestingly, DOX promotes the early phase of autophagy initiation but inhibits the late phase by blocking autophagosome and lysosome fusion and lysosome acidification [42]. The inconsistencies observed in the literature regarding DOX can be attributed to various factors such as experimental models, dosing, and treatment duration [43]. Additionally, conflicting findings in DOX autophagy research can be influenced by the methods used to monitor autophagy, including the specific targets examined and the interpretation of autophagic flux as a flowchart process rather than as snapshots of some instances.

Several studies have delved into the role of autophagy in metabolic cardiomyopathy. Research has shown that the natural process of autophagy is disrupted in the hearts of individuals with diabetes [44]. However, the specific impact of autophagy on diabetic cardiomyopathy is still not fully understood. Researchers have found evidence suggesting that autophagy may have both protective and harmful effects in diabetic cardiomyopathy [45,46], indicating that its role is multifaceted. The findings of most of these studies indicate that metabolic alterations hinder cardiac autophagy and mitophagy, with deleterious consequences [47,48,49,50]. The study by Tong and colleagues discovered that although mitophagy is activated during the early stages of diabetes in a mouse model of high-fat-diet-induced cardiomyopathy, it is insufficient to shield the heart. Moreover, improving mitochondrial function and preserving cardiac function requires enhancing mitophagy [50].

## 3. Dietary Flavonoids Are Capable of Modulating Autophagy in Cardiomyocytes

**Figure 2:**
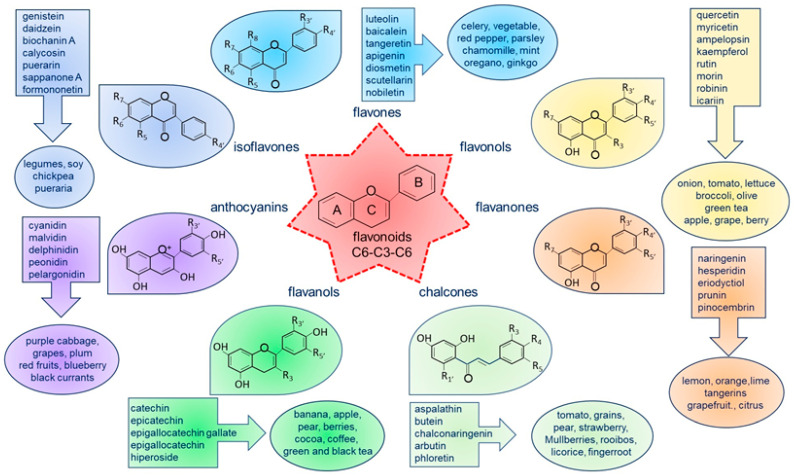
Basic molecular structure of flavonoids, classification, and examples of natural food sources and their distribution in various plants. The classes of flavonoids are color-coded to show their natural distribution, along with examples of the most common compounds found in the diet.

Flavonoids have recently been recognized as agents capable of modulating autophagy, thus improving cell homeostasis and function [51]. Flavonoids are a large class of low-molecular-weight natural compounds [52,53]. They are widely present in diverse edible and medicinal plants, such as vegetables (onion, celery, parsley, broccoli, spinach, and beans), fruits (apple, grape, oranges, lemon, mandarin, cherry, and berries), seeds (coffee and cocoa), grains, herbs (black and green tea) [54], wine [55], and food (cocoa, chocolate). Flavonoids contribute to plants’ color, fragrance, and flavor characteristics while also serving various essential functions, such as attracting pollinators, regulating cell growth, and protecting against stresses. The basic chemical structure of flavonoids consists of a 15-carbon skeleton—also known as C6-C3-C6 carbon skeleton—containing two phenolic benzene rings (A and B) bridged by a heterocyclic pyran or pyrone (C). Flavonoids are classified into seven subclasses based on a modification of their bare skeletons: flavones, flavonols, flavanones, chalcones, catechins or flavanols, anthocyanins, and isoflavones [56], as shown in Figure 2.

Epidemiological evidence suggests that increased intake of flavonoids not only inhibits tumor formation and inflammation but also may be linked to a reduced risk of developing CVD [57,58,59,60]. In the early 1990s, a series of prospective studies involving humans were initiated, and over the past thirty years, more than 40 publications have studied the impact of flavonoid intake on CVD risk. Despite some disparities in the findings across these studies, comprehensive meta-analyses have consistently suggested a strong positive correlation between the habitual, long-term consumption of flavonoid-rich diets and a decreased risk of ischemic heart disease, cerebrovascular disease, and total CVD [61].

In recent decades, various flavonoids—particularly those found in ordinary dietary sources—have been extensively studied for their potential cardioprotective effects in preclinical models [62,63,64]. In most of these studies, the focus has been on the antioxidant and anti-inflammatory properties of flavonoids, which may be the basis of their cardioprotective effect. However, in this paper, we want to focus on the effect of flavonoids on autophagy. In the following sections, we will analyze the studies on the effects of flavonoids on autophagy, categorizing them based on the specific cardiac diseases being investigated.

### 3.1. The Effects of Flavonoids on Autophagy in Ischemia and I/R Injury

An imbalance between oxygen demand and supply, known as ischemia, occurs when there is reduced or no blood flow to the heart muscle, leading to damage or dysfunction of the cardiac tissue and causing a myocardial infarction. Ischemia becomes chronic when blood flow is not restored promptly. On the other hand, timely restoration of blood flow, known as reperfusion, is the most effective strategy for reducing the size of an infarct and improving the clinical outcome. However, reintroducing blood flow to the ischemic heart can also cause injury, which is referred to as reperfusion injury. This injury results from complex mechanisms involving the production of reactive oxygen species, changes in intracellular calcium handling, dysfunction of small blood vessels and endothelial cells, altered heart muscle metabolism, and activation of inflammation.

Table 1 summarizes preclinical studies that delved into flavonoid effects on autophagy in both in vivo and in vitro cardiac models of ischemic and ischemia/reperfusion damage. In all the studies reviewed, the different subgroups of flavonoids investigated exhibited cardioprotective effects on ischemic-based cardiac impairment. However, their impact on autophagy was highly heterogeneous.

Four studies have investigated the effects of different flavone compounds (apigenin, luteolin, nobiletin, and scutellarin) on both in vivo and in vitro models of mice or rats. These flavones have been shown to enhance autophagy induced by ischemia or I/R [65,66,67,68] in both acute and long-term phases. In particular, to investigate the beneficial acute effects of flavones, Wang et al. [67] and Xu et al. [68] proposed I/R models for mice and rats, respectively, focusing on the acute response of the infarcted area. The first research group administered apigenin at the reperfusion and stopped the mice after 48 h; the latter group administered scutellarin 15 min before ischemia and stopped the rats 24 h after reperfusion. Similarly, the treatment schedule of cell cultures also followed the scheme of the respective experimental designs. Regardless of the administration method or analysis time used, the positive results on cardiac readouts selected by the authors were consistent with an improvement and linked to an increase in autophagy during the acute phases. Additionally, two other studies were conducted by Hu et al. [65] and Wu et al. [66], who investigated chronic ischemia (MI) models in mice and rats, evaluating the effect of flavone administration on the remodeling process 4 and 3 weeks after coronary occlusion, respectively. It is worth noting that in the experimental designs of [65,66], there was a difference in the timing of the administration of the compounds being studied. The former group administered luteolin as a prophylactic pretreatment 3 days before the occlusion of the artery, while the latter researchers administered nobiletin as a therapeutic treatment 1 day after the occlusion when some of the apoptotic and necrotic events had already taken place. The second design is more relevant in a therapeutic translational context. Although the readouts were partly different in the two studies, the results proved positive in both cases and were associated with increased autophagy.

Two studies have investigated the impact of isoflavones, specifically formononetin and sappanone A, on an ex vivo model of rodent hearts isolated and perfused in the Langendorff apparatus [69,70]. The model used was a nonworking beating heart in which global no-flow ischemia was induced, followed by reperfusion. The study by Huang and coworkers involved administering the flavonoid post-conditioning, which means short administrations repeated during total reperfusion. Meanwhile, in the study by Shi and colleagues, the authors administered sappanone A in the first 15 min of reperfusion. In both cases, the researchers investigated the effect of the isoflavones on acute reperfusion injury. In both cases, the effect on acute reperfusion injury (maximum 90 min) was mitigated, and autophagy was increased.

Otherwise, anthocyanin (cyanidin-3-O-glucoside) and flavanone (hesperidin) were found to reduce autophagy to achieve cardioprotection [71,72]. The study conducted by Shan and collaborators found reduced autophagy and reduced acute cardiac damage when cyanidin-3-O-glucoside was administered as a prophylactic treatment for one week before inducing I/R in rats. A similar outcome was also observed in their in vitro model of aged H9c2 cells subjected to oxygen and glucose deprivation, mimicking the effects of ischemia, followed by 24 h of reperfusion in the presence of anthocyanin [71]. Additionally, flavanone hesperidin reduced I/R injury in the rat hearts when given as prophylactic treatment for 3 days while also decreasing autophagy [72].

It is interesting to note that two different flavanols, epigallocatechin gallate (EGG) [73,74] and hiperoside [75], were tested for their cardioprotective effects on rat hearts. However, they had opposite outcomes in autophagy. EGG was used in two different in vivo acute I/R studies and was administered 10 min before reperfusion [73] or 30 min before ischemia [74], resulting in fewer cardiac injuries and reduced levels of autophagy. Zhang and colleagues reproduced similar results in their H9c2 in vitro models by incubating cells with EGG 4 h before hypoxia/reperfusion [74].

Differently, Yang and colleagues [75] conducted a study in mice using a chronic ischemia model to investigate the effect of hiperoside on ventricular remodeling. They began the therapeutic treatment after the onset of the infarction. The flavanol used in the treatment effectively reduced the infarct area and circulating damage biomarkers. However, the results were associated with an increase in autophagy.

**Table 1:** Effects of flavonoids on myocardial autophagy in myocardial ischemia and I/R injury.

Compound	Animal/ Cell Line	Model	Flavonoid Dose	Effect on the Heart/Cells	Auto-Phagy	Ref
Apigenin *(flavone)*	Mouse (C57BL6)	In vivo I/R (30 min/48 h)	40 mg/kg iv at reperfusion	**↓** Infarct size	**↑**	[67]
	Mouse cardiomyocytes	In vitro H/R	100 μM incubated after H/R	**↑** Mitochondria function **↓** Apoptosis	**↑**	
Luteolin *(flavone)*	Mouse (C57BL6)	In vivo MI (4-wk ischemia)	10 µg/kg ip for 3 d prior MI	**↑** LV function **↓** Apoptosis Serum LDH, IL-1α, MPO CK, TNF-α	**↑**	[65]
	Mouse neonatal cardiomyocytes	In vitro hypoxia (8 h)	8 µM 48 h prior hypoxia	**↑** Mitochondria function	**↑**	
Nobiletin *(flavone)*	Rat (Sprague-Dawley)	In vivo MI (3-wk ischemia)	5 mg/kg/d ip 1 d after MI	**↓** Mortality **↑** LV function **↓** Infarct size, fibrosis	**↑**	[66]
	H9c2	in vitro ischemia OGD (12 h)	20 µM 2 h prior OGD	**↓** Apoptosis	**↑**	
Scutellarin *(flavone)*	Rat (Sprague-Dawley)	In vivo I/R (30 min/24 h)	10–20 mg/kg ip 15 min prior ischemia	**↓** Infarct size, apoptosis **↑** LV function	**↑**	[68]
	H9c2	In vitro OGD/R (6 h/24 h)	6.25–12.5 mg/mL 6 h prior OGD	**↓** Apoptosis, LDH release	**↑**	
Formononetin *(isoflavone)*	Mouse	Ex vivo Langendorff no flow I/R (40 min/60 min)	5 mM for 10 s at reperfusion, repeated 5 times over 60 min	**↑** LV function **↓** Infarct size, apoptosis	**↑**	[69]
	H9c2 (aged cells)	In vitro H/R	5 mM	**↓** Apoptosis	**↑**	
Sappanone A *(isoflavone)*	Rat (Wistar)	Ex vivo Langendorff no flow I/R (30 min/90 min)	100 μM in the first 15 min of reperfusion	**↑** Mitochondria function and quality control	**↑**	[70]
Epigallocatechin gallate *(flavanol)*	Rat (Sprague-Dawley)	In vivo I/R (30 min/2 h)	10 mg/kg iv 10 min prior reperfusion	**↑** LV function **↓** infarct size, apoptosis, serum CK and LDH	**↓**	[73]
Epigallocatechin gallate *(flavanol)*	Rat (Sprague-Dawley)	In vivo I/R (30 min/12 h)	10 mg/kg iv 30 min prior ischemia	**↓** Infarct size, serum cTnI	**↓**	[74]
	H9c2	In vitro H/R (6 h/12 h)	25 μM incubated 4 h prior H/R	**↓** cTnI release	**↓**	
Hiperoside (*flavanol)*	Mouse (Kunming)	In vivo MI (2-wk ischemia)	9–36 mg/kg/d ig for 2 wk after MI	**↑** LV function **↓** Fibrosis **↓** Serum CK, cTnI, LDH	**↑**	[75]
Hesperidin *(flavanone)*	Rat (Sprague-Dawley)	In vivo I/R (30 min/4 h)	200 mg/kg/d ig for 3 d prior I/R	**↓** Infarct size, serum CK, and cTnI	**↓**	[72]
Cyanidin-3-glucoside *(anthocyanin)*	Rat (Sprague-Dawley)	In vivo I/R (30 min/2 h)	10–20 mg/kg/d ip for 7d prior I/R	**↓** Infarct size, tissue damage, and ferroptosis	**↓**	[71]
	H9c2	In vitro OGD/R (6 h/24 h)	25–100 μM incubated during OGD/R	**↓** ROS, ferroptosis	**↓**	

I/R: ischemia/reperfusion; cTnI: cardiac troponin I; H/R: hypoxia/reoxygenation; MI: myocardial infarction; LV: left ventricle; CK: creatine kinase; LDH: lactate dehydrogenase; IL-1α: interleukin 1α; MPO: myeloperoxidase; TNF-α: Tumor necrosis factor-α; OGD: oxygen-glucose deprivation; OGD/R: oxygen-glucose deprivation/reoxygenation; ROS: reactive oxygen species; iv: intravenous; ip: intraperitoneal; ig: intragastric. Upward arrow (**↑**) indicates an increase, while a downward arrow (**↓**) indicates a decrease.

It is important to note that due to the diverse design and type of studies analyzed, comparing and drawing conclusions from the authors’ findings is challenging. These studies had varying objectives, such as selecting an ischemia or ischemia/reperfusion damage model, timing of the investigation, type of treatment (prophylactic or therapeutic), and route of flavonoid administration, as shown in Table 1. Consequently, the criteria used to evaluate performance were also diverse and differed significantly.

### 3.2. The Effects of Flavonoids on Autophagy in Doxorubicin Cardiotoxicity

The impact of flavonoids on autophagy in models of chemotherapy-induced cardiotoxicity was investigated in both in vitro and in vivo studies, as detailed in Table 2.

In 5 out of 12 (42%) of the studies, researchers investigated the effects of flavonols [76,77,78,79,80], while 25% of the studies focused on the impact of flavones [81,82,83] or isoflavones [84,85,86], and only one investigation used a chalcone flavonoid [87]. All classes of flavonoids examined were found to be effective in reducing DOX cardiotoxicity by improving cardiac function and reducing cell damage or apoptosis. In various studies, the cardiotoxic effects of DOX were linked to either an increase or decrease in autophagy. Similarly, the impact of different flavonoids, while consistently associated with improved cardiotoxicity, yielded conflicting results regarding their effect on autophagy in different studies. In all in vitro studies, treatment with flavonoids occurred before and/or in combination with DOX. Out of eight in vivo studies, four coadministered the flavonoid compound with DOX treatment for varying durations and cumulative doses, analyzing either acute or chronic effects [80,81,83,85]. In three other studies, the experimental design involved administering the flavonoid as a pretreatment, followed by continued administration in combination with DOX [76,79,86].

**Table 2:** Effects of flavonoids on myocardial autophagy in preclinical DOX-induced cardiotoxicity.

Compound	Animal/ Cell Line	Model	Flavonoid Dose	Time of Analysis	Effect on the Heart/Cells	Auto-phagy	Ref
Apigenin *(flavone)*	Mouse (Kunming)	In vivo	125–250 mg/kg/d ig with DOX for 16 d	1 d after reaching DOX cumulative dose (24 mg/kg)	**↓** Apoptosis Serum AST, CK, LDH activity	**↓**	[81]
Luteolin *(flavone)*	Adult mouse cardiomyocytes	In vitro	1–50 μM incubated with DOX	24 h after DOX + Luteolin incubation	**↑** Contractile function **↓** Apoptosis and ROS	**↑**	[82]
Scutellarin *(flavone)*	Rat	In vivo	10 mg/kg/d ip with DOX for 6 wk	2 wk after reaching DOX cumulative dose (20 mg/kg)	**↑** LV function **↓** cTnT, fibrosis Apoptosis	**↓**	[83]
Calycosin *(isoflavone)*	Zebrafish adult	In vivo	5 μmol/L in water for 4 wk after 4 wk from DOX injection	8 wk after a single DOX injection	**↑** LV function **↓** Natriuretic peptides	**↑**	[84]
Daidzein *(isoflavone)*	Rat (Sprague-Dawley)	In vivo	20 mg/kg/d sc prior weekly DOX	6 wk after DOX initiation	**↑** LV function **↓** Apoptosis	**↓**	[86]
Puerarin *(isoflavone)*	Mouse	In vivo	100 mg/kg with DOX	3 wk to reach DOX cumulative dose (15 mg/kg)	**↑** LV function **↓** Inflammation, tissue damage	**↑**	[85]
	H9c2	In vitro	80–320 μM incubated 24 h prior DOX	48 h post DOX exposure	**↑** Viability, mitochondria function **↓** ROS, LDH release	**↑**	
Ampelopsin *(flavonol)*	Mouse (C57BL6)	In vivo	50–100 mg/kg ig 24 h prior and during DOX, daily over 12 d	24 h after reaching the DOX cumulative dose (15 mg/kg)	**↑** LV function **↓** Fibrosis and apoptosis	**↑**	[76]
Icariin *(flavonol)*	H9c2	In vitro	1–5 μM incubated 3 h prior DOX	24 h after DOX incubation	**↑** Viability **↓** Apoptosis, ROS	**↓**	[77]
Rosa roxburghii *(flavanol + flavonol)*	Rat neonatal cardiomyocytes + H9c2	In vitro	40–80 μg/mL incubated 12–36 h prior DOX	12 h after DOX incubation	**↓** Morphological changes	**↓**	[78]
Rutin *(flavonol)*	Mouse (C57BL6)	In vivo	100 mg/kg os from 6 d prior DOX to sacrifice	8 wk after reaching DOX cumulative dose (21 mg/kg)	**↑** LV function **↓** Fibrosis and apoptosis	**↓**	[79]
	Rat neonatal cardiomyocytes	In vitro	10 μM (24 h pretreatment + 24 h with DOX)	24 h after DOX exposure	**↓** Apoptosis	**↓**	
Spinacetin *(flavonol)*	Rat (Sprague-Dawley)	In vivo + in vitro	50–100 mg/kg ig concomitant to DOX for 14 d	24 h after the last DOX injection	**↑** Survival rate **↓** Apoptosis, injury markers	**↑**	[80]
Aspalathin *(chalcone)*	H9c2	In vitro	0.2 μM incubated with DOX	After 5 d of DOX + Aspalathin exposure	**↓** Apoptosis	**↑**	[87]

DOX: doxorubicin; AST: aspartate aminotransferase; CK: creatine kinase; LDH: lactate dehydrogenase; ROS: reactive oxygen species; cTnT: cardiac troponin T; LV: left ventricle; ig: intragastric; ip: intraperitoneal; sc: subcutaneous; os: oral. Upward arrow (**↑**) indicates an increase, while a downward arrow (**↓**) indicates a decrease.

Oxidative/nitrosative stress in cardiomyocytes plays a primary role in DOX-induced cardiotoxicity. DOX can trigger the cardiac production of large quantities of reactive oxygen and nitrogen species [88]. It is possible that pretreatment or simultaneous administration of the flavonoid with the chemotherapeutic agent might prevent the toxic action of DOX rather than rescue and reverse the cardiotoxicity. Regrettably, the sole study that administered flavonoids after the onset of DOX-induced cardiotoxicity utilized zebrafish as the animal model [84]. This makes it less comparable to other data in the literature.

### 3.3. The Effects of Flavonoids on Hypertrophic Cardiomyopathy

Pathological remodeling of the ventricle associated with hypertrophic growth is a critical step in the development of heart failure and has been linked to dysregulation of the autophagic process [89,90]. Therefore, there is a growing interest in exploring the various properties of flavonoids to target this pathological remodeling. Table 3 summarizes the in vivo and in vitro studies that investigated the impact of flavonoids on autophagy in models of cardiac hypertrophy.

In all the studies and preclinical models used, flavonoids have demonstrated a cardioprotective effect by modulating autophagy in the opposite direction to that triggered by the model under examination.

Regarding in vivo models, different forms of cardiac stress to trigger hypertrophic remodeling have been used. In two rat studies, a pressure overload model was used through thoracic aorta constriction [91,92], while in a mouse study, a neurohormonal cardiac stress model was used through adrenergic hyperstimulation [93]. In all cases, an increase in autophagy was observed with all classes of flavonoids tested (flavone, flavanol, and isoflavone).

**Table 3:** Effects of flavonoids on myocardial autophagy in hypertrophic cardiomyopathy.

Compound	Animal/ Cell Line	Model	Flavonoid Dose	Effect on the Heart/Cells	Auto-Phagy	Ref
Baicalein *(flavone)*	Mouse (C57BL6)	In vivo (ISO-induced hypertrophy)	25 mg/kg iv every 3 d during 15 d daily ISO treatment	**↑** LV function **↓** Hypertrophic markers	**↑**	[93]
	Rat neonatal cardiomyocytes	In vitro (ISO-induced hypertrophy)	30 μM 4 h prior 24 h ISO incubation	**↓** Cell surface, hypertrophic markers	**↑**	
Diosmetin *(flavone)*	Rat neonatal cardiomyocytes	In vitro (PE-induced hypertrophy)	10 -50 μM coincubated with PE for 12–24 h	**↓** Cell surface, hypertrophic markers	**↓**	[94]
Luteolin *(flavone)*	Rat neonatal cardiomyocytes	In vitro (LPS-induced hypertrophy)	50–100 mg/mL coincubated with LPS for 8 h	**↑** Cell viability **↓** Cell surface, hypertrophic markers	**↓**	[95]
Hiperoside (*flavanol)*	Rat (Wistar)	In vivo (TAC-induced hypertrophy)	200 mg/kg/d ig after TAC for 6 wk	**↑** LV function **↓** Hypertrophy, CSA, apoptosis	**↑**	[92]
	H9C2	In vitro (AngII-induced hypertrophy	10 μM coincubated with AngII for 48 h	**↓** Apoptosis	**↑**	
Puerarin *(isoflavone)*	Rat (Sprague-Dawley)	In vivo (TAC- induced hypertrophy)	100 mg/kg/d sc at TAC for 6 wk	**↑** LV function **↓** Hypertrophic markers, CSA, apoptosis	**↑**	[91]
	H9c2	In vitro (ISO-induced hypertrophy)	20 µM 24 h prior ISO followed by incubation with ISO for 6 h	**↓** Cell surface, hypertrophic markers, apoptosis	**↑**	

ISO: isoproterenol; LV: left ventricle; PE: phenylephrine; LPS: lipopolysaccharide; TAC: thoracic aorta constriction; CSA: cross-sectional area (cardiomyocytes); AngII: angiotensin II; iv: intravenous; ig: intragastric; sc: subcutaneous. Upward arrow (**↑**) indicates an increase, while a downward arrow (**↓**) indicates a decrease.

Out of the five in vitro studies, four employed a cellular model to replicate hypertrophy resulting from neurohormonal stimulation, specifically adrenergic hyperstimulation [91,92,93,94]. The remaining study used a model that simulated neurohormonal stimulation through increased calcium signaling induced by lipopolysaccharide injection [94]. However, the findings regarding the effects of flavonoids were inconsistent, both across different classes of flavonoids and within the same class. Specifically, among the flavones examined on neonatal rat cardiomyocytes, baicalein increased the decreased autophagy caused by the stressful stimulus [93], while diosmetin and luteolin reduced the autophagy induced by the hypertrophic stimulus [94,95]. Additionally, both the flavanol hiperoside and the isoflavone puerarin, tested on H9c2 cells, demonstrated an increase in autophagy that had been reduced by neurohormonal stimulation [91,92].

### 3.4. The Effects of Flavonoids on Autophagy in Other Cardiomyopathies

Rodents that show signs of prediabetes and type II diabetes, along with diet-induced obesity, are commonly employed to study the hypoglycemic effects of flavonoids. Beyond their effects on metabolic disorders, numerous studies have also examined how flavonoids affect cardiometabolic diseases by studying the process of autophagy. Table 4 provides an overview of in vivo studies focused on animal models of type II diabetes.

Flavonoids consistently exhibit a protective effect on the heart compared to changes caused by diabetes. Additionally, they have opposite effects on autophagy compared to the effects observed in the untreated pathological model. All studies were conducted on rodents, with five studies on rats [96,97,98,99,100] and one on mice [101]. In all studies except one, diabetes was induced using streptozotocin (STZ), a compound that preferentially targets pancreatic β cells, in the absence [100,101] or presence of a high lipid and/or carbohydrate diet [97,98,99]. However, Liu and colleagues used the Goto–Kakizaki (GK) rat [96]. In the GK, diabetes-related traits are controlled by multiple genes, and different genetic loci regulate glucose tolerance, insulin secretion, β-cell mass, and plasma lipids.

After the onset of diabetic pathology, flavonoid treatment was administered in most studies, with the exception of one study by Su and colleagues [99], where treatment was administered as diabetes developed. Among the classes of flavonoids investigated, the flavonol ampelosin (also known as dihydromyricetin) was found to increase autophagy [97], while luteolin, a flavone, was associated with a reduction in autophagy [100], and scutellarin, another flavone, led to an increase in autophagy [99]. Notably, conflicting results were observed with the flavanol epigallocatechin gallate in two different studies [96,98], possibly due to differences in the diabetic etiologies of the models used.

Table 3 also displays the in vivo and in vitro studies that examined the impact of flavonoids on the autophagic process in models of septic cardiomyopathy. Sepsis is a widespread inflammatory response that occurs after a bacterial infection. Cardiac dysfunction is a significant result of sepsis, affecting mortality and being linked to either heightened inflammation or the suppression of both fatty acid and glucose oxidation, leading to ATP depletion.

In experiments conducted in animals, researchers have found that flavonoids, specifically flavones, can effectively reduce dysfunction and damage in the heart muscle [102,103,104]. However, the timing of administration, the specific rodent species used in the studies, and the effects on autophagy varied among different research investigations.

Apigenin and luteolin increased autophagy in mice, with apigenin administered immediately after sepsis induction and luteolin administered within 10 days of pretreatment [102,104]. In contrast, tangeretin, administered in rats concomitantly with the induction of sepsis, showed a reduction in autophagy [103]. Pretreatment with the isoflavone puerarin in H9c2 cells also reduced apoptosis, ROS production, and limited mitochondrial damage in association with increased autophagy [105].

**Table 4:** Effects of flavonoids on autophagy in preclinical cardiotoxicity induced by diabetes or sepsis.

Compound	Animal/ Cell Line	Model	Flavonoid Dose	Effect on the Heart/Cells	Auto-Phagy	Ref
***Type II Diabetes***						
Ampelopsin *(flavonol)*	Rat (Wistar)	In vivo (STZ + diet cardiomyopathy)	100 mg/kg/d 3 wk after diabetes induction for 15 wk	**↑** LV function **↓** Fibrosis, apoptosis	**↑**	[97]
Ampelopsin *(flavonol)*	Mouse (C57BL6)	In vivo (STZ cardiomyopathy)	100 mg/kg/d ig 3 wk after diabetes induction for 14 wk	**↑** LV function **↓** Fibrosis, apoptosis, inflammation, oxidative markers	**↑**	[101]
Epigallocatechin gallate *(flavanol)*	Rat (Goto–Kakizaki, spontaneous model of diabetes)	In vivo (cardiomyopathy)	100 mg/kg/d ig for 6 wk	**↑** Mitochondrial function **↓** Oxidative stress	**↓**	[96]
Epigallocatechin gallate *(flavanol)*	Rat (Sprague-Dawley)	In vivo (HFD + STZ cardiomyopathy)	40–80 mg/kg ig for 8 wk after diabetes assessment	**↑** LV function **↓** Injury markers, fibrosis	**↑**	[98]
Luteolin *(flavone)*	Rat (Sprague-Dawley)	In vivo (STZ cardiomyopathy)	50–200 mg/kg ig for 4 wk after 6 wk of diabetes assessment	**↑** LV function **↓** Fibrosis	**↓**	[100]
Scutellarin *(flavone)*	Rat (Sprague-Dawley)	In vivo (STZ + diet cardiomyopathy)	100–200 mg/kg/d for 8 wk during diabetes onset	**↓** Hypertrophy, LDH, CK release, apoptosis	**↑**	[99]
***Sepsis***						
Apigenin *(flavone)*	Mouse (C57BL6)	In vivo (endotoxin-induced cardiomyopathy)	50 mg/kg ip 1 h post sepsis induction	**↑** LV function **↓** Apoptosis, cardiac damage, LDH, CK release	**↑**	[102]
Luteolin *(flavone)*	Mouse (C57BL6)	In vivo (endotoxin-induced cardiomyopathy)	10 μg/kg ip 10 d prior to sepsis induction	**↑** LV function **↑** Mitochondrial function, **↓** Apoptosis, inflammatory markers	**↑**	[104]
Tangeretin *(flavone)*	Rat (Sprague-Dawley)	In vivo (endotoxin-induced cardiomyopathy)	50–100 mg/kg os concomitant with sepsis induction for 24 h	**↓** Apoptosis, oxidative markers, cTnI, cMLC1 release, inflammatory infiltration	**↓**	[103]
Puerarin *(isoflavone)*	H9c2	In vitro (endotoxin-induced cardiomyopathy)	100 mg/l 24 h prior LPS incubation for 24 h	**↓** Apoptosis, ROS, mitochondrial injury	**↑**	[105]

STZ: streptozotocin; LV: left ventricle; HFD: high-fat diet; LPS: lipopolysaccharide; CK: creatine kinase; LDH: lactate dehydrogenase; cTnI: cardiac troponin I; cMLC1: cardiac myosin-light chains 1; LPS: lipopolysaccharide; ROS: reactive oxygen species; ig: intragastric; ip: intraperitoneal; os: oral. Upward arrow (**↑**) indicates an increase, while a downward arrow (**↓**) indicates a decrease.

## 4. Discussion

### 4.1. Lessons Learned from Preclinical Studies and Potential Critical Issues for the Translational Perspective

The extensive body of evidence emphasized by the above-cited preclinical studies clearly shows that several flavonoids positively impact cardiac pathologies of various etiologies, partially through the regulation of autophagy.

However, when considering the potential application of these findings in a clinical context, it is important to reflect on certain key aspects. Many mechanistic studies, especially those conducted in vitro, have exposed cells to unusually high concentrations of various flavonoids. Additionally, in some in vivo studies, flavonoids were administered through intravenous or intraperitoneal routes, which differs significantly from the natural intake of flavonoids through diet.

Findings derived from supraphysiological doses provide valuable insights into the potential therapeutic applications of flavonoids. However, their relevance to the cardiometabolic effects of flavonoids at typical dietary intake levels is limited. Another important consideration when interpreting data from preclinical studies is that any route of administration for flavonoids different from the dietary or oral ones underestimates the complexity of their bioavailability and metabolism [106]. Bioavailability varies significantly among different flavonoids but is, in general, very low [107]. Therefore, the most abundant ones in our diet may not necessarily result in the highest concentrations of active metabolites in target tissues. In addition, food composition and flavonoid source are likely to affect bioavailability. Moreover, most dietary flavonoids are in the form of glycosides, which are not effectively absorbed by the body compared to aglycones. Upon consumption, only about 10% of flavonoid glycosides are absorbed in the upper gastrointestinal tract. The remaining 90% travel through the small intestine to eventually reach the colon as unmetabolized and unabsorbed flavonoids [108]. They need to be broken down through hydrolysis before they can be absorbed in the intestine [109]. After absorption, flavonoids undergo phase I and phase II metabolism in the liver, where they are converted into hydroxylated, glucuronidated, sulfated, and methylated metabolites [110]. These metabolites exhibit longer half-lives and reach higher circulating concentrations than the parent flavonoid compounds. Therefore, it is challenging to determine the specific beneficial effects of individual metabolites compared to the original flavonoid consumed through diet. Additionally, except for a small portion of flavonoid glycosides, which are hydrolyzed by digestive enzymes, most of the unabsorbed flavonoids are transformed in aglycones by the gut microbiota [110,111]. In a study, Warner and colleagues found that microbiome-derived phenolic metabolites of flavonoids reduced the secretion of adhesion molecules in human endothelial cell cultures compared with their parent compounds [112]. Emerging evidence suggests that flavonoid consumption and gut microbiota interact bidirectionally, impacting physiological pathways relevant to cardiometabolic health. Flavonoids have been reported to increase the proportion of beneficial bacteria phyla in the intestine, thus regulating and balancing the disordered microbiota [113,114,115].

### 4.2. Autophagy as a Cellular Target of Flavonoids in Cardiovascular Diseases

It is worth noting that analyzed publications indicated that the balance between cell death and survival, influenced by changes in autophagy, plays an important role in the pathophysiology of a number of cardiomyopathies. As mentioned above, autophagy is not absolutely beneficial in the occurrence of cardiovascular diseases. It is like a “double-edged sword” with a dual role [4]. On the one hand, physiological autophagy has a protective effect on the maintenance of cardiovascular function. On the other hand, overexpression of autophagy can also induce cell death, so as to promote the development of disease. In view of this controversy, it is particularly important to regulate autophagy at a reasonable rate of activity. As a consequence, the cardioprotective impact of flavonoids is achieved through bidirectional regulation of autophagic targets. For example, studies on apigenin, luteolin, and scutellarin have shown contradictory outcomes (see Table 1, Table 2, Table 3 and Table 4). This could be attributed to differences in experimental conditions as well as the specific type of autophagy involved in the mechanisms of cardiac damage caused by different pathophysiological conditions associated with the type of cardiomyopathy. It is now known that alternative pathways regulating autophagy exist. However, further investigations are needed to deepen the research of autophagic mechanisms and better dissect the regulatory signals underlying this paradoxical consequence.

The method used to evaluate the autophagic process is also crucial and can lead to unclear or inconsistent results. The autophagic process is complex, involving several phases, each of which should be examined with appropriate markers. For the process to work correctly, there must be a continuous operational flow (autophagic flux) involving all stages and components, similar to a flow chart. Dysregulation of the process, as seen in various CVD conditions, can occur in one or more stages, such as the initial phases of autophagosome formation or the more advanced stages of lysosome–autophagosome fusion, ultimately resulting in a blockage of the autophagic flux. The beneficial effect of flavonoids can be exerted in one or more stages, depending on the type of flavonoid and the specific type of alteration present. Recent studies on preclinical models of pathophysiological conditions other than CVD have highlighted the ability of flavonoids to re-establish the autophagic process by promoting autophagosome–lysosome fusion as one of the key targetable steps through a complete analysis of the autophagic process [116,117,118,119]. The selected studies in this paper clearly demonstrate the link between flavonoids and the regulation of the autophagic process. It is important to note, however, that flavonoids have various properties, such as antioxidant and anti-inflammatory activities [53]. It cannot be ruled out that these properties may have contributed to the observed cardioprotective effects. In particular, inflammation not only represents a significant risk factor for CVD but also plays a crucial role as a treatable outcome of the disease. Moreover, inflammation is widely acknowledged to have a substantial impact on regulating the adverse left ventricular remodeling that follows a myocardial infarction. As a result, flavonoids have been recognized for their essential cardioprotective properties, attributed to their ability to target multiple inflammatory mediators in response to I/R-induced inflammation [68,120,121,122,123].

Additionally, while studies have dissected the role of individual flavonoids in a diet rich in fruits and vegetables, multiple flavonoids can be consumed. Therefore, further studies are needed to explore the possible interactions, synergies, or interferences of multiple flavonoids taken together.

## 5. Conclusions

As foods containing flavonoids are widely consumed and their mechanisms of action are still only partially understood, this review compares the effects and mechanisms of action of some of the most widely characterized dietary flavonoids in the treatment of autophagy-deregulation-related cardiac diseases. Flavonoids could potentially constitute important adjuvant agents of conventional therapies to treat autophagy-related cardiomyopathies. Since the majority of people consume flavonoids on a regular basis, their effect on human health is significant, and improving our knowledge of the complex mechanisms underlying the bioactivity of flavonoids may accelerate the development of dietary data-supported interventions. In conclusion, this research offers insights for future research on the cardioprotective potential of flavonoids mediated by the modulation of autophagic processes, highlighting the need for further in-depth research to fully exploit their positive impact on human health.

## Abbreviations

AMP	adenosine monophosphate
AMPK	AMP-activated kinase
AngII	angiotensin II
AST	aspartate aminotransferase
ATG	autophagy-related
ATP	adenosine triphosphate
BCL2L13	BCL2 Like 13
BNIP3	BCL-2/adenovirus E1B 19kD protein-interacting protein 3
CK	creatine kinase
cMLC1	cardiac myosin-light chains 1
CSA	cross-sectional area
cTnI	cardiac troponin I
cTnT	cardiac troponin T
CVD	cardiovascular disease
DOX	doxorubicin
EGG	epigallocatechin gallate
FUNDC1	FUN14 Domain Containing 1
GABA	gamma-aminobutyric acid
GABARAP	GABA type A receptor-associated protein
GK	Goto–Kakizaki
HFD	high-fat diet
H/R	hypoxia/reoxygenation
HSC70	heat shock cognate 70 protein
IL-1α	interleukin 1α
I/R	ischemia/reperfusion
ig	intragastric
ip	intraperitoneal
ISO	isoproterenol
iv	intravenous
LAMP-2A	lysosomal-associated membrane protein-2A
LDH	lactate dehydrogenase
LIR	LC3-interacting region
LPS	lipopolysaccharide
LV	left ventricle
MAP1LC3/LC3	microtubule-associated protein 1 light chain 3
MI	myocardial infarction
MPO	myeloperoxidase
mTOR	mechanistic target of rapamycin
OGD	oxygen-glucose deprivation
OGD/R	oxygen-glucose deprivation/reoxygenation
OPTN	optineurin
os	oral
OXPHOS	oxidative phosphorylation
Parkin	Parkin RBR E3 ubiquitin-protein ligase
PE	phenylephrine
PINK1	PTEN-induced kinase 1
ROS	reactive oxygen species
sc	subcutaneous
STZ	streptozotocin
TAC	thoracic aorta constriction
TNF-α	Tumor necrosis factor-α
ULK-1	Unc-51 kinase-like-1

## References

[1] Global Nutrition Report (2021). 2021 Global Nutrition Report: The State of Global Nutrition. https://globalnutritionreport.org/reports/2021-global-nutrition-report/.

[2] Mente A., Dehghan M., Rangarajan S., O’Donnell M., Hu W., Dagenais G., Wielgosz A., Lear S.A., Wei L., Diaz R. (2023). Diet, cardiovascular disease, and mortality in 80 countries. Eur. Heart J..

[3] Mozaffarian D., Wu J.H.Y. (2018). Flavonoids, dairy foods, and cardiovascular and metabolic health. A review of emerging biologic pathways. Circ. Res..

[4] Jiang B., Zhou X., Yang T., Wang L., Feng L., Wang Z., Xu J., Jing W., Wang T., Su H. (2023). The role of autophagy in cardiovascular disease: Cross-interference of signaling pathways and underlying therapeutic targets. Front. Cardiovasc. Med..

[5] Weiss R.G., Gerstenblith G., Bottomley P.A. (2005). ATP flux through creatine kinase in the normal, stressed, and failing human heart. Proc. Natl. Acad. Sci. USA.

[6] Ploumi C., Daskalaki I., Tavernarakis N. (2017). Mitochondrial biogenesis and clearance: A balancing act. FEBS J..

[7] Ding Q., Qi Y., Tsang S.-Y. (2021). Mitochondrial biogenesis, mitochondrial dynamics, and mitophagy in the maturation of cardiomyocytes. Cells.

[8] He C., Klionsky D.J. (2009). Regulation mechanisms and signaling pathways of autophagy. Annu. Rev. Genet..

[9] Klionsky D.J., Petroni G., Amaravadi R.K., Baehrecke E.H., Ballabio A., Boya P., Bravo-San Pedro J.M., Cadwell K., Cecconi F., Choi A.M.K. (2021). Autophagy in major human diseases. EMBO J..

[10] Schuck S. (2020). Microautophagy–distinct molecular mechanisms handle cargoes of many sizes. J. Cell Sci..

[11] Rios J., Sequeida A., Albornoz A., Budini M. (2021). Chaperone mediated autophagy substrates and components in cancer. Front. Oncol..

[12] Johansen T., Lamark T. (2020). Selective autophagy: ATG8 family proteins, LIR motifs and cargo receptors. J. Mol. Biol..

[13] Germain K., Kim P.K. (2020). Pexophagy: A model for selective autophagy. Int. J. Mol. Sci..

[14] Kravic B., Behrends C., Meyer H. (2020). Regulation of lysosome integrity and lysophagy by the ubiquitin-conjugating enzyme UBE2QL1. Autophagy.

[15] Li W., He P., Huang Y., Li Y.F., Lu J., Li M., Kurihara H., Luo Z., Meng T., Onishi M. (2021). Selective autophagy of intracellular organelles: Recent research advances. Theranostics.

[16] Bauer B., Martens S., Ferrari L. (2023). Aggrephagy at a glance. J. Cell Sci..

[17] Vargas J.N.S., Hamasaki M., Kawabata T., Youle R.J., Yoshimori T. (2023). The mechanisms and roles of selective autophagy in mammals. Nat. Rev. Mol. Cell Biol..

[18] Dikic I., Elazar Z. (2018). Mechanism and medical implications of mammalian autophagy. Nat. Rev. Mol. Cell Biol..

[19] Galluzzi L., Pietrocola F., Levine B., Kroemer G. (2014). Metabolic control of autophagy. Cell.

[20] Hansen M., Rubinsztein D.C., Walker D.W. (2018). Autophagy as a promoter of longevity: Insights from model organisms. Nat. Rev. Mol. Cell Biol..

[21] Mizushima N., Yoshimori T., Ohsumi Y. (2011). The role of Atg proteins in autophagosome formation. Annu. Rev. Cell Dev. Biol..

[22] Egan D., Kim J., Shaw R.J., Guan K.L. (2011). The autophagy initiating kinase ULK1 is regulated via opposing phosphorylation by AMPK and mTOR. Autophagy.

[23] Kirkin V., Rogov V.V. (2019). A diversity of selective autophagy receptors determines the specificity of the autophagy pathway. Mol. Cell.

[24] Gubas A., Dikic I. (2022). A guide to the regulation of selective autophagy receptors. FEBS J..

[25] Tong M., Sadoshima J. (2016). Mitochondrial autophagy in cardiomyopathy. Curr. Opin. Genet. Dev..

[26] Bravo-San Pedro J.M., Kroemer G., Galluzzi L. (2017). Autophagy and mitophagy in cardiovascular disease. Circ. Res..

[27] Sciarretta S., Maejima Y., Zablocki D., Sadoshima J. (2018). The role of autophagy in the heart. Annu. Rev. Physiol..

[28] Ikeda Y., Shirakabe A., Maejima Y., Zhai P., Sciarretta S., Toli J., Nomura M., Mihara K., Egashira K., Ohishi M. (2015). Endogenous Drp1 mediates mitochondrial autophagy and protects the heart against energy stress. Circ. Res..

[29] Shirakabe A., Zhai P., Ikeda Y., Saito T., Maejima Y., Hsu C.P., Nomura M., Egashira K., Levine B., Sadoshima J. (2016). Drp1-dependent mitochondrial autophagy plays a protective role against pressure overload-induced mitochondrial dysfunction and heart failure. Circulation.

[30] Kaludercic N., Maiuri M.C., Kaushik S., Fernández Á.F., de Bruijn J., Castoldi F., Chen Y., Ito J., Mukai R., Murakawa T. (2020). Comprehensive autophagy evaluation in cardiac disease models. Cardiovasc. Res..

[31] Kanamori H., Takemura G., Goto K., Maruyama R., Tsujimoto A., Ogino A., Takeyama T., Kawaguchi T., Watanabe T., Fujiwara T. (2011). The role of autophagy emerging in postinfarction cardiac remodelling. Cardiovasc. Res..

[32] Kubli D.A., Zhang X., Lee Y., Hanna R.A., Quinsay M.N., Nguyen C.K., Jimenez R., Petrosyan S., Murphy A.N., Gustafsson A.B. (2013). Parkin protein deficiency exacerbates cardiac injury and reduces survival following myocardial infarction. J. Biol. Chem..

[33] Wang Y., Jasper H., Toan S., Muid D., Chang X., Zhou H. (2021). Mitophagy coordinates the mitochondrial unfolded protein response to attenuate inflammation-mediated myocardial injury. Redox Biol..

[34] Matsui Y., Takagi H., Qu X., Abdellatif M., Sakoda H., Asano T., Levine B., Sadoshima J. (2007). Distinct roles of autophagy in the heart during ischemia and reperfusion: Roles of AMP-activated protein kinase and Beclin 1 in mediating autophagy. Circ. Res..

[35] Zhai P., Sciarretta S., Galeotti J., Volpe M., Sadoshima J. (2011). Differential roles of GSK-3beta during myocardial ischemia and ischemia/reperfusion. Circ. Res..

[36] Xie M., Kong Y., Tan W., May H., Battiprolu P.K., Pedrozo Z., Wang Z.V., Morales C., Luo X., Cho G. (2014). Histone deacetylase inhibition blunts ischemia/reperfusion injury by inducing cardiomyocyte autophagy. Circulation.

[37] Dimitrakis P., Romay-Ogando M.I., Timolati F., Suter T.M., Zuppinger C. (2012). Effects of doxorubicin cancer therapy on autophagy and the ubiquitin-proteasome system in long-term cultured adult rat cardiomyocytes. Cell Tissue Res..

[38] Wang X., Wang X.L., Chen H.L., Wu D., Chen J.X., Wang X.X., Li R.L., He J.H., Mo L., Cen X. (2014). Ghrelin inhibits doxorubicin cardiotoxicity by inhibiting excessive autophagy through AMPK and p38-MAPK. Biochem. Pharmacol..

[39] Xu X., Chen K., Kobayashi S., Timm D., Liang Q. (2012). Resveratrol attenuates doxorubicin-induced cardiomyocyte death via inhibition of p70 S6 kinase 1-mediated autophagy. J. Pharmacol. Exp. Ther..

[40] Kawaguchi T., Takemura G., Kanamori H., Takeyama T., Watanabe T., Morishita K., Ogino A., Tsujimoto A., Goto K., Maruyama R. (2012). Prior starvation mitigates acute doxorubicin cardiotoxicity through restoration of autophagy in affected cardiomyocytes. Cardiovasc. Res..

[41] Sishi B.J., Loos B., van Rooyen J., Engelbrecht A.M. (2013). Autophagy upregulation promotes survival and attenuates doxorubicin-induced cardiotoxicity. Biochem. Pharmacol..

[42] Koleini N., Kardami E. (2017). Autophagy and mitophagy in the context of doxorubicin-induced cardiotoxicity. Oncotarget.

[43] Dirks-Naylor A.J. (2013). The role of autophagy in doxorubicin-induced cardiotoxicity. Life Sci..

[44] Dewanjee S., Vallamkondu J., Kalra R.S., John A., Reddy P.H., Kandimalla R. (2021). Autophagy in the diabetic heart: A potential pharmacotherapeutic target in diabetic cardiomyopathy. Ageing Res. Rev..

[45] Xu T., Ding W., Ji X., Ao X., Liu Y., Yu W., Wang J. (2019). Oxidative stress in cell death and cardiovascular diseases. Oxid. Med. Cell. Longev..

[46] Luo J., Yan D., Li S., Liu S., Zeng F., Cheung C.W., Liu H., Irwin M.G., Huang H., Xia Z. (2020). Allopurinol reduces oxidative stress and activates Nrf2/p62 to attenuate diabetic cardiomyopathy in rats. J. Cell. Mol. Med..

[47] Xie Z., He C., Zou M.H. (2011). AMP-activated protein kinase modulates cardiac autophagy in diabetic cardiomyopathy. Autophagy.

[48] Li Z.L., Woollard J.R., Ebrahimi B., Crane J.A., Jordan K.L., Lerman A., Wang S.M., Lerman L.O. (2012). Transition from obesity to metabolic syndrome is associated with altered myocardial autophagy and apoptosis. Arterioscler. Thromb. Vasc. Biol..

[49] Sciarretta S., Boppana V.S., Umapathi M., Frati G., Sadoshima J. (2015). Boosting autophagy in the diabetic heart: A translational perspective. Cardiovasc. Diagn. Ther..

[50] Tong M., Saito T., Zhai P., Oka S., Mizushima W., Nakamura M., Ikeda S., Shirakabe A., Sadoshima J. (2019). Mitophagy is essential for maintaining cardiac function during high fat diet-induced diabetic cardiomyopathy. Circ. Res..

[51] D’Arcy M.S. (2022). A review of biologically active flavonoids as inducers of autophagy and apoptosis in neoplastic cells and as cytoprotective agents in non-neoplastic cells. Cell Biol. Int..

[52] Santos E.L., Maia B., Ferriani A.P., Teixeira S.D. (2017). Flavonoids: Classification, biosynthesis and chemical ecology. Flavonoids: From Biosynthesis to Human Health.

[53] Dias M.C., Pinto D.C.G.A., Silva A.M.S. (2021). Plant flavonoids: Chemical characteristics and biological activity. Molecules.

[54] Testai L. (2015). Flavonoids and mitochondrial pharmacology: A new paradigm for cardioprotection. Life Sci..

[55] Somerset S.M., Johannot L. (2008). Dietary flavonoid sources in Australian adults. Nutr. Cancer.

[56] Chen S., Wang X., Cheng Y., Gao H., Chen X. (2023). A review of classification, biosynthesis, biological activities and potential applications of flavonoids. Molecules.

[57] Ros E., Martínez-González M.A., Estruch R., Salas-Salvadó J., Fitó M., Martínez J.A., Corella D. (2014). Mediterranean diet and cardiovascular health: Teachings of the PREDIMED Study. Adv. Nutr..

[58] Lapuente M., Estruch R., Shahbaz M., Casas R. (2019). Relation of fruits and vegetables with major cardiometabolic risk factors, markers of oxidation, and inflammation. Nutrients.

[59] Ciumărnean L., Milaciu M.V., Runcan O., Vesa Ș.C., Răchișan A.L., Negrean V., Perné M.G., Donca V.I., Alexescu T.G., Para I. (2020). The effects of flavonoids in cardiovascular diseases. Molecules.

[60] Ullah A., Munir S., Badshah S.L., Khan N., Ghani L., Poulson B.G., Emwas A.H., Jaremko M. (2020). Important flavonoids and their role as a therapeutic agent. Molecules.

[61] Parmenter B.H., Croft K.D., Hodgson J.M., Dalgaard F., Bondonno C.P., Lewis J.R., Cassidy A., Scalbert A., Bondonno N.P. (2020). An overview and update on the epidemiology of flavonoid intake and cardiovascular disease risk. Food Funct..

[62] Allawadhi P., Khurana A., Sayed N., Kumari P., Godugu C. (2018). Isoproterenol-induced cardiac ischemia and fibrosis: Plant-based approaches for intervention. Phytother. Res..

[63] Viswanatha G.L., Shylaja H., Keni R., Nandakumar K., Rajesh S. (2022). A systematic review and meta-analysis on the cardio-protective activity of naringin based on pre-clinical evidences. Phytother. Res..

[64] Zhang W., Zheng Y., Yan F., Dong M., Ren Y. (2023). Research progress of quercetin in cardiovascular disease. Front. Cardiovasc. Med..

[65] Hu J., Man W., Shen M., Zhang M., Lin J., Wang T., Duan Y., Li C., Zhang R., Gao E. (2016). Luteolin alleviates post-infarction cardiac dysfunction by up-regulating autophagy through Mst1 inhibition. J. Cell. Mol. Med..

[66] Wu X., Zheng D., Qin Y., Liu Z., Zhang G., Zhu X., Zeng L., Liang Z. (2017). Nobiletin attenuates adverse cardiac remodeling after acute myocardial infarction in rats via restoring autophagy flux. Biochem. Biophys. Res. Commun..

[67] Wang Z., Zhang H., Liu Z., Ma Z., An D., Xu D. (2020). Apigenin attenuates myocardial infarction-induced cardiomyocyte injury by modulating Parkin-mediated mitochondrial autophagy. J. Biosci..

[68] Xu L.J., Chen R.C., Ma X.Y., Zhu Y., Sun G.B., Sun X.B. (2020). Scutellarin protects against myocardial ischemia-reperfusion injury by suppressing NLRP3 inflammasome activation. Phytomedicine.

[69] Huang Z., Liu Y., Huang X. (2018). Formononetin may protect aged hearts from ischemia/reperfusion damage by enhancing autophagic degradation. Mol. Med. Rep..

[70] Shi X., Li Y., Wang Y., Ding T., Zhang X., Wu N. (2021). Pharmacological postconditioning with sappanone A ameliorates myocardial ischemia reperfusion injury and mitochondrial dysfunction via AMPK-mediated mitochondrial quality control. Toxicol. Appl. Pharmacol..

[71] Shan X., Lv Z.Y., Yin M.J., Chen J., Wang J., Wu Q.N. (2021). The Protective effect of cyanidin-3-glucoside on myocardial ischemia-reperfusion injury through ferroptosis. Oxid. Med. Cell Longev..

[72] Li X., Hu X., Wang J., Xu W., Yi H., Ma R., Jiang H. (2018). Inhibition of autophagy via activation of PI3K/Akt/mTOR pathway contributes to the protection of hesperidin against myocardial ischemia/reperfusion injury. Int. J. Mol. Med..

[73] Xuan F., Jian J. (2016). Epigallocatechin gallate exerts protective effects against myocardial ischemia/reperfusion injury through the PI3K/Akt pathway-mediated inhibition of apoptosis and the restoration of the autophagic flux. Int. J. Mol. Med..

[74] Zhang C., Liang R., Gan X., Yang X., Chen L., Jian J. (2019). MicroRNA-384-5p/Beclin-1 as potential indicators for Epigallocatechin Gallate against cardiomyocytes ischemia reperfusion injury by inhibiting autophagy via PI3K/Akt pathway. Drug Des. Dev. Ther..

[75] Yang Y., Li J., Rao T., Fang Z., Zhang J. (2021). The role and mechanism of hyperoside against myocardial infarction in mice by regulating autophagy via NLRP1 inflammation pathway. J. Ethnopharmacol..

[76] Li X., Wang X., Wang B., Chi W., Li Z., Zhang M., Shen Y., Liu X., Lu Y., Liu Y. (2022). Dihydromyricetin protects against Doxorubicin-induced cardiotoxicity through activation of AMPK/mTOR pathway. Phytomedicine.

[77] Scicchitano M., Carresi C., Nucera S., Ruga S., Maiuolo J., Macrì R., Scarano F., Bosco F., Mollace R., Cardamone A. (2021). Icariin protects H9c2 rat cardiomyoblasts from Doxorubicin-induced cardiotoxicity: Role of caveolin-1 upregulation and enhanced autophagic response. Nutrients.

[78] Yuan H., Wang Y., Chen H., Cai X. (2020). Protective effect of flavonoids from Rosa roxburghii Tratt on myocardial cells via autophagy. 3 Biotech.

[79] Ma Y., Yang L., Ma J., Lu L., Wang X., Ren J., Yang J. (2017). Rutin attenuates doxorubicin-induced cardiotoxicity via regulating autophagy and apoptosis. Biochim. Biophys. Acta Mol. Basis Dis..

[80] Liu D., Zhao L. (2022). Spinacetin alleviates doxorubicin-induced cardiotoxicity by initiating protective autophagy through SIRT3/AMPK/mTOR pathways. Phytomedicine.

[81] Yu W., Sun H., Zha W., Cui W., Xu L., Min Q., Wu J. (2017). Apigenin attenuates adriamycin-induced cardiomyocyte apoptosis via the PI3K/AKT/mTOR pathway. Evid. Based Complement. Alternat. Med..

[82] Xu H., Yu W., Sun S., Li C., Zhang Y., Ren J. (2020). Luteolin attenuates doxorubicin-induced cardiotoxicity through promoting mitochondrial autophagy. Front. Physiol..

[83] Sun X., Zhou L., Han Y., Yang Q., Li X., Xin B., Chi M., Wang Y., Guo C. (2023). Scutellarin attenuates doxorubicin-induced cardiotoxicity by inhibiting myocardial fibrosis, apoptosis and autophagy in rats. Chem. Biodivers..

[84] Lu X., Lu L., Gao L., Wang Y., Wang W. (2021). Calycosin attenuates doxorubicin-induced cardiotoxicity via autophagy regulation in zebrafish models. Biomed. Pharmacother..

[85] Peng Y., Wang L., Zhang Z., He X., Fan Q., Cheng X., Qiao Y., Huang H., Lai S., Wan Q. (2022). Puerarin activates adaptive autophagy and protects the myocardium against doxorubicin-induced cardiotoxicity via the 14-3-3gamma/PKCepsilon pathway. Biomed. Pharmacother..

[86] Wu J., Li K., Liu Y., Feng A., Liu C., Adu-Amankwaah J., Ji M., Ma Y., Hao Y., Bu H. (2023). Daidzein ameliorates doxorubicin-induced cardiac injury by inhibiting autophagy and apoptosis in rats. Food Funct..

[87] Johnson R., Shabalala S., Louw J., Kappo A.P., Muller C.J.F. (2017). Aspalathin reverts doxorubicin-induced cardiotoxicity through in-creased autophagy and decreased expression of p53/mTOR/p62signaling. Molecules.

[88] Songbo M., Lang H., Xinyong C., Bin X., Ping Z., Liang S. (2019). Oxidative stress injury in doxorubicin-induced cardiotoxicity. Toxicol. Lett..

[89] Gu J., Hu W., Song Z.P., Chen Y.G., Zhang D.D., Wang C.Q. (2016). Rapamycin inhibits cardiac hypertrophy by promoting autophagy via the MEK/ERK/Beclin-1 pathway. Front. Physiol..

[90] Lu J., Sun D., Liu Z., Li M., Hong H., Liu C., Gao S., Li H., Cai Y., Chen S. (2016). SIRT6 suppresses isoproterenol-induced cardiac hypertrophy through activation of autophagy. Transl. Res..

[91] Liu B., Wu Z., Li Y., Ou C., Huang Z., Zhang J., Liu P., Luo C., Chen M. (2015). Puerarin prevents cardiac hypertrophy induced by pressure overload through activation of autophagy. Biochem. Biophys. Res. Commun..

[92] Guo X., Zhang Y., Lu C., Qu F., Jiang X. (2020). Protective effect of hyperoside on heart failure rats via attenuating myocardial apoptosis and inducing autophagy. Biosci. Biotechnol. Biochem..

[93] Liu B., Li L., Liu G., Ding W., Chang W., Xu T., Ji X., Zheng X., Zhang J., Wang J. (2021). Baicalein attenuates cardiac hypertrophy in mice via suppressing oxidative stress and activating autophagy in cardiomyocytes. Acta Pharmacol. Sin..

[94] Guo Y., Li D., Cen X.F., Qiu H.L., Ma Y.L., Liu Y., Huang S.H., Liu L.B., Xu M., Tang Q.Z. (2022). Diosmetin protects against cardiac hypertrophy via p62/Keap1/Nrf2 signaling pathway. Oxid. Med. Cell Longev..

[95] Li X., Liu J., Wang J., Zhang D. (2019). Luteolin suppresses lipopolysaccharide-induced cardiomyocyte hypertrophy and autophagy in vitro. Mol. Med. Rep..

[96] Liu J., Tang Y., Feng Z., Long J. (2014). (–)-Epigallocatechin-3-gallate attenuated myocardial mitochondrial dysfunction and autophagy in diabetic Goto–Kakizaki rats. Free Radic. Res..

[97] Ni T., Lin N., Lu W., Sun Z., Lin H., Chi J., Guo H. (2020). Dihydromyricetin prevents diabetic cardiomyopathy via miR-34a suppression by activating autophagy. Cardiovasc. Drugs Ther..

[98] Jia Q., Yang R., Mehmood S., Li Y. (2022). Epigallocatechin-3-gallate attenuates myocardial fibrosis in diabetic rats by activating autophagy. Exp. Biol. Med..

[99] Su Y., Fan X., Li S., Li Z., Tian M., Li S. (2022). Scutellarin improves type 2 diabetic cardiomyopathy by regulating cardiomyocyte autophagy and apoptosis. Dis. Markers.

[100] Xiao C., Chen M.Y., Han Y.P., Liu L.J., Yan J.L., Qian L.B. (2023). The protection of luteolin against diabetic cardiomyopathy in rats is related to reversing JNK-suppressed autophagy. Food Funct..

[101] Wu B., Lin J., Luo J., Han D., Fan M., Guo T., Tao L., Yuan M., Yi F. (2017). Dihydromyricetin protects against diabetic cardiomyopathy in Streptozotocin-induced diabetic mice. Biomed. Res. Int..

[102] Li F., Lang F., Zhang H., Xu L., Wang Y., Zhai C., Hao E. (2017). Apigenin alleviates endotoxin-induced myocardial toxicity by modulating inflammation, oxidative stress, and autophagy. Oxid. Med. Cell Longev..

[103] Shiroorkar P.N., Afzal O., Kazmi I., Al-Abbas F.A., Altamimi A.S.A., Gubbiyappa K.S., Sreeharsha N. (2020). Cardioprotective effect of tangeretin by inhibiting PTEN/AKT/mTOR axis in experimental sepsis-induced myocardial dysfunction. Molecules.

[104] Wu B., Song H., Fan M., You F., Zhang L., Luo J., Li J., Wang L., Li C., Yuan M. (2020). Luteolin attenuates sepsis-induced myocardial injury by enhancing autophagy in mice. Int. J. Mol. Med..

[105] Chang X., He Y., Wang L., Luo C., Liu Y., Li R. (2022). Puerarin alleviates LPS-induced H9C2 cell injury by inducing mitochondrial autophagy. J. Cardiovasc. Pharmacol..

[106] Manach C., Williamson G., Morand C., Scalbert A., Rémésy C. (2005). Bioavailability and bioefficacy of polyphenols in humans. I. Review of 97 bioavailability studies. Am. J. Clin. Nutr..

[107] Gonzales G.B., Smagghe G., Grootaert C., Zotti M., Raes K., Van Camp J. (2015). Flavonoid interactions during digestion, absorption, distribution and metabolism: A sequential structure-activity/property relationship-based approach in the study of bioavailability and bioactivity. Drug Metab. Rev..

[108] Pei R., Liu X., Bolling B. (2020). Flavonoids and gut health. Curr. Opin. Biotechnol..

[109] Chuankayan P., Rimlumduan T., Svasti J., Ketudat Cairns J.R. (2007). Hydrolysis of soybean isoflavonoid glycosides by dalbergia β-glucosidases. J. Agric. Food Chem..

[110] Cassidy A., Minihane A.M. (2017). The role of metabolism (and the microbiome) in defining the clinical efficacy of dietary flavonoids. Am. J. Clin. Nutr..

[111] Stevens Y., Rymenant E.V., Grootaert C., Camp J.V., Possemiers S., Masclee A., Jonkers D. (2019). The intestinal fate of citrus flavanones and their effects on gastrointestinal health. Nutrients.

[112] Warner E.F., Zhang Q., Raheem K.S., O’Hagan D., O’Connell M.A., Kay C.D. (2016). Common phenolic metabolites of flavonoids, but not their unmetabolized precursors, reduce the secretion of vascular cellular adhesion molecules by human endothelial cells. J. Nutr..

[113] Estruel-Amades S., Massot-Cladera M., Pérez-Cano F.J., Franch À., Castell M., Camps-Bossacoma M. (2019). Hesperidin effects on gut microbiota and gut-associated lymphoid tissue in healthy rats. Nutrients.

[114] Li Q., Gao B., Siqin B., He Q., Zhang R., Meng X., Zhang N., Zhang N., Li M. (2021). Gut microbiota: A novel regulator of cardiovascular disease and key factor in the therapeutic effects of flavonoids. Front. Pharmacol..

[115] Pan L., Ye H., Pi X., Liu W., Wang Z., Zhang Y., Zheng J. (2023). Effects of several flavonoids on human gut microbiota and its metabolism by in vitro simulated fermentation. Front. Microbiol..

[116] Hu P., Qing Y., Li H., Sun W., Yu X., Hui H., Guo Q., Xu J. (2021). FV-429 induces autophagy blockage and lysosome-dependent cell death of T-cell malignancies via lysosomal dysregulation. Cell Death Dis..

[117] Zhao Y., Li Z.F., Zhang D., Wang Z.Y., Wang L. (2021). Quercetin alleviates Cadmium-induced autophagy inhibition via TFEB-dependent lysosomal restoration in primary proximal tubular cells. Ecotoxicol. Environ. Saf..

[118] Liu H., Zhou W., Guo L., Zhang H., Guan L., Yan X., Zhai Y., Qiao Y., Wang Z., Zhao J. (2022). Quercetin protects against palmitate-induced pancreatic β-cell apoptosis by restoring lysosomal function and autophagic flux. J. Nutr. Biochem..

[119] Guan L., Guo L., Zhang H., Liu H., Zhou W., Zhai Y., Yan X., Men X., Peng L. (2024). Naringin protects against non-alcoholic fatty liver disease by promoting autophagic flux and lipophagy. Mol. Nutr. Food Res..

[120] Ma C., Jiang Y., Zhang X., Chen X., Liu Z., Tian X. (2018). Isoquercetin ameliorates myocardial infarction through anti-inflammation and anti-apoptosis factor and regulating TLR4-NF-κB signal pathway. Mol. Med. Rep..

[121] Wang Z.K., Chen R.R., Li J.H., Chen J.Y., Li W., Niu X.L., Wang F.F., Wang J., Yang J.X. (2020). Puerarin protects against myocardial ischemia/reperfusion injury by inhibiting inflammation and the NLRP3 inflammasome: The role of the SIRT1/NF-kappaB pathway. Int. Immunopharmacol..

[122] Zhao L., Zhou Z., Zhu C., Fu Z., Yu D. (2020). Luteolin alleviates myocardial ischemia reperfusion injury in rats via Siti1/NLRP3/NF-κB pathway. Int. Immunopharmacol..

[123] Liu W., Cheng L., Li X., Zhao L., Hu X., Ma Z. (2022). Short-term pretreatment of naringin isolated from Citrus wilsonii Tanaka attenuates rat myocardial ischemia/reperfusion injury. Naunyn Schmiedeb. Arch. Pharmacol..

